# Safety and efficacy of dexmedetomidine in interventional chest procedures: A systematic review

**DOI:** 10.1097/MD.0000000000043911

**Published:** 2025-08-15

**Authors:** Abdulrhman M. Khaity, Nada M. Al-Dardery, Mohamed El-Samahy, Moaz M. El-Sayed, Sheikh M. Jamal, Kalpana Singh, Abdulqadir J. Nashwan

**Affiliations:** a Faculty of Medicine, Elrazi University, Khartoum, Sudan; b Faculty of Medicine, Fayoum University, Fayoum, Egypt; c Faculty of Medicine, Zagazig University, Zagazig, Egypt; d Internal Medicine Department, Hazm Mebaireek General Hospital, Doha, Qatar; e Nursing & Midwifery Research Department (NMRD), Hamad Medical Corporation, Doha, Qatar; f Department of Public Health, College of Health Sciences, QU Health, Qatar University, Doha, Qatar.

**Keywords:** dexmedetomidine, medical thoracoscopy, systematic review

## Abstract

**Background:**

Interventional chest procedures, including medical thoracoscopy (MT) and video-assisted thoracoscopic surgery (VATS), are integral to diagnosing and treating thoracic conditions. Dexmedetomidine is increasingly used for sedation in these procedures due to its favorable pharmacological profile, yet its comparative efficacy and safety remain inadequately defined. This systematic review aims to comprehensively summarize the available evidence on the efficacy and safety of dexmedetomidine for sedation during MT and VATS.

**Methods:**

Following PRISMA guidelines, we searched 4 electronic databases (PubMed, Web of Science, Cochrane Library, Scopus) for studies published until August 2024. We included randomized controlled trials (RCTs) and observational studies that assessed dexmedetomidine in patients undergoing MT or VATS. Outcomes measured included pain scores, postoperative complications, and lengths of hospital or care unit stay.

**Results:**

Seven studies (six RCTs and one prospective cohort study) involving a total of 442 patients were included. Dexmedetomidine demonstrated enhanced analgesic efficacy in comparison to midazolam across multiple investigations. Although dexmedetomidine has shown promise in reducing intensive care unit durations in certain studies, its influence on overall hospital length of stay exhibited variability. Adverse events were largely comparable between the 2 sedatives, although instances of hypotension were noted more frequently with dexmedetomidine in some comparisons.

**Conclusion:**

Ultimately, dexmedetomidine has emerged as a reliable sedative option for thoracoscopic interventions, providing efficient pain relief while maintaining stable respiratory parameters. Nonetheless, its cardiovascular ramifications warrant vigilant oversight. This review highlights the imperative for additional standardized research to validate these outcomes and inform evidence-based sedation protocols.

## 1. Introduction

Medical thoracoscopy (MT) and video-assisted thoracoscopic surgery (VATS) are vital procedures for both diagnostic and therapeutic management of pleural and parenchymal lung diseases.^[1,2]^ Pleuroscopy, often used interchangeably with MT, refers to a minimally invasive thoracoscopic technique performed under procedural sedation in spontaneously breathing patients.^[3,4]^ It is especially valuable for evaluating unexplained exudative pleural effusions, offering a higher diagnostic yield compared to percutaneous needle biopsy.^[3,4]^ VATS, a form of thoracoscopic surgery, is typically performed under general anesthesia with endotracheal intubation and one-lung ventilation and is considered a more invasive approach.^[2]^

Both MT and VATS require carefully selected sedation to reduce patient discomfort and anxiety, which can otherwise compromise procedural success.^[5–7]^ The choice of sedative agents, such as midazolam and dexmedetomidine, is critical to ensuring patient comfort, cooperation, and optimal procedural outcomes.^[7–9]^

Dexmedetomidine, a highly selective α2-adrenoreceptor agonist, has emerged as a valuable sedative agent due to its unique pharmacologic profile. It produces a sedation state that closely mimics natural sleep while preserving patient arousability and cooperation, particularly beneficial during procedures requiring patient responsiveness, such as MT and VATS.^[8–10]^ Moreover, dexmedetomidine is associated with minimal respiratory depression and a relatively stable hemodynamic profile, making it a favorable option for patients with underlying pulmonary or cardiovascular risk factors.^[11]^

Despite its increasing use in procedural sedation, the current literature evaluating dexmedetomidine in chest interventions remains scattered and heterogeneous. While some studies have examined its role in VATS and MT, a comprehensive synthesis of outcomes such as procedural safety, patient cooperation, pain control, and recovery quality is lacking.^[12–15]^ Variability in sedation protocols and patient populations across studies further complicates interpretation.^[10–15]^ Thus, there is a clear need for a systematic review to consolidate the available evidence and inform sedation strategies in interventional chest procedures, particularly for high-risk cohorts.

In this systematic review, we aim to comprehensively summarize the safety and efficacy of dexmedetomidine in terms of pain control, procedural complications, and lengths of hospital or care unit stay for patients undergoing MT and VATS. By summarizing the existing evidence about the clinical relevance of dexmedetomidine, we can guide clinicians in making evidence-based choices to enhance patient safety, procedural efficiency, and overall outcomes in MT and VATS.

## 2. Methods

The PRISMA guidelines and cochrane collaboration recommendations were used to prepare this review.^[16]^ All steps of this study were prespecified, and the protocol was registered on PROSPERO (CRD42024549530).

### 2.1. Search strategy

We searched 4 electronic medical databases: PubMed, Web of Science, Cochrane Library, and Scopus from inception until August 2024 using the following query: (Midazolam OR Dexmedetomidine) AND (thoracoscopy OR thoracoscopic OR pleuroscopy).

### 2.2. Study eligibility

Studies were included if they met the following criteria:

*Population:* patients undergoing interventional chest procedures, including MT and VATS.

*Intervention groups:* Studies that evaluated dexmedetomidine as a sedative agent, either used alone or in comparison to other agents such as midazolam (with or without fentanyl), normal saline, or oxycodone.

*Outcomes:* Pain score, postoperative medical or surgical complications, and lengths of hospital or care unit stay.

*Study design:* All full-text randomized controlled trials (RCTs) or observational studies

We excluded case reports, case series, conference abstracts, and non-English studies. We also excluded studies that did not include dexmedetomidine as a sedative agent during MT or VATS, or that did not assess the target outcomes of interest.

### 2.3. Selection of studies

Three reviewers independently screened the titles and abstracts of all retrieved citations to assess their relevance for inclusion. Full-text articles of potentially eligible studies were then reviewed in detail to determine their suitability for the systematic review. The citation screening process was facilitated using Rayyan software. Discrepancies among reviewers were resolved through discussion with a fourth independent author.

### 2.4. Data extraction

Two reviewers independently extracted key data from each included study using a standardized, pilot-tested data extraction form. Extracted information included study characteristics, population demographics, relevant clinical outcomes such as pain assessment, postoperative medical or surgical complications, and the lengths of hospital or care unit stay (days). Summary of findings and risk of bias assessments were also recorded. Any discrepancies in data extraction were resolved by discussion with a third reviewer.

### 2.5. Quality appraisal

Two reviewers independently assessed the methodological quality of included studies. For RCTs, the cochrane risk of bias (ROB 2) Tool^[17]^ was used, while the Newcastle-Ottawa scale^[18]^ was applied for observational studies. Any disagreements in assessment were resolved through discussion with a third reviewer.

### 2.6. Data synthesis

Due to significant clinical and methodological heterogeneity across the included studies, such as variation in interventions, comparators, outcome measures, and patient populations, a meta-analysis was not feasible. As such, we conducted a systematic review without meta-analysis, synthesizing findings descriptively for each outcome of interest.^[19,20]^

## 3. Results

### 3.1. Results of literature search and study characteristics

Our search strategy identified 3565 studies from 4 electronic databases. After removing duplicates, 2187 abstracts were screened, and 19 articles underwent full-text evaluation. Of these, 12 were excluded (5 for not meeting outcomes, 3 as reviews, and 4 for unrelated interventions). Seven studies,^[21–27]^ (6 RCTs and one prospective cohort), were included in this systematic review, totaling 442 participants. Five studies involved VATS, while 2 focused on MT.

**Figure 1.**
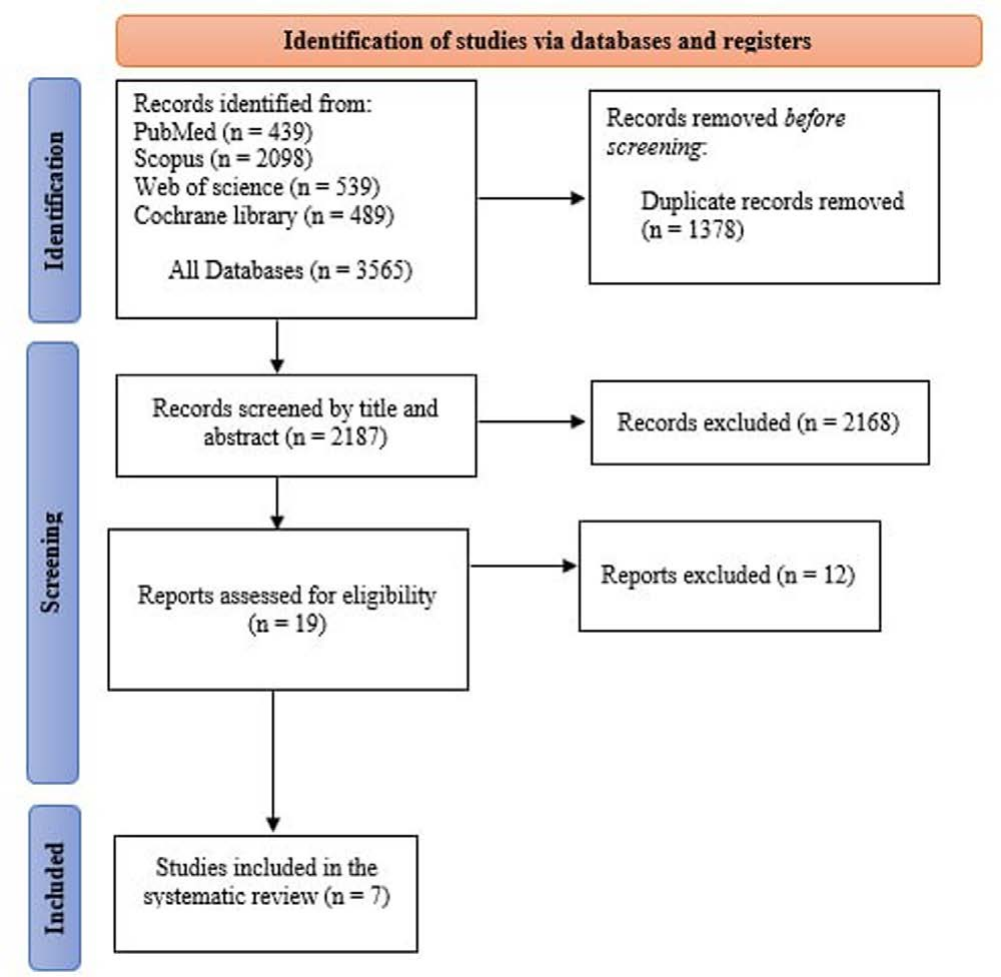
PRISMA flow diagram of studies’ screening and selection.

**Figure 2.**
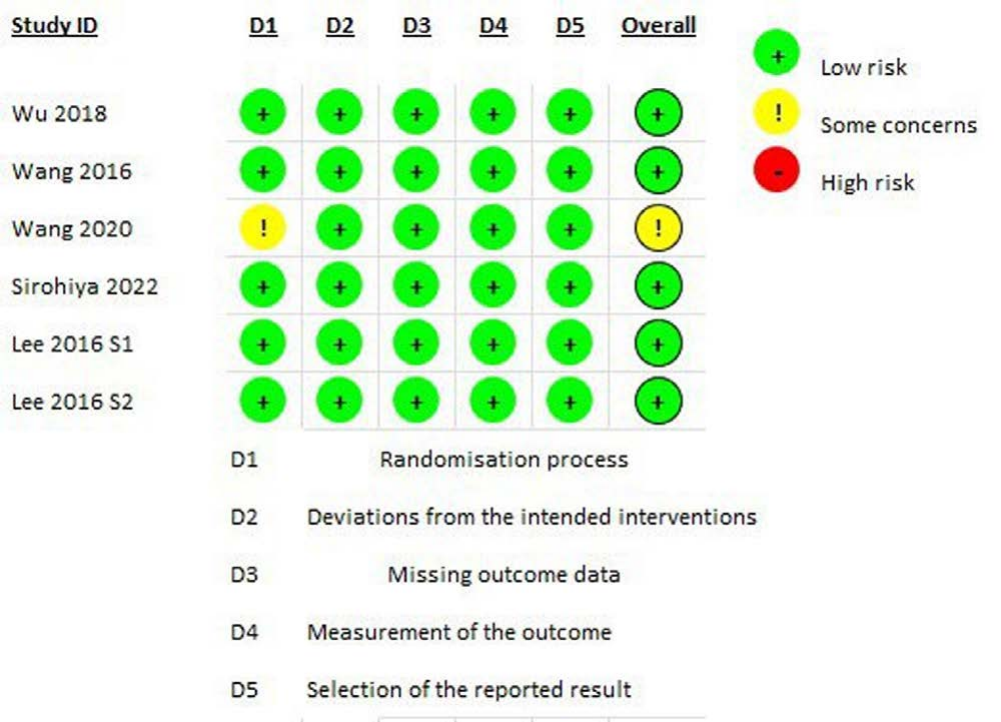
The risk of bias summary and risk of bias graph.

The PRISMA flowchart depicting the study selection methodology is illustrated in Figure 1. A summary of the included studies, their design, baseline characteristics, and main results is shown in Table 1. The quality of the studies was assessed using ROB2, which showed that 6 studies had a low risk of bias (Figure 2). The Newcastle-Ottawa scale revealed that the cohort study had a good quality score (Table S1, Supplemental Digital Content, https://links.lww.com/MD/P692).

**Table 1:** Summary and baseline characteristics of the included studies.

Study ID	Design	Sedative agents used	Sample size, n	Age, yr, mean (SD)	Sex, male (%)	BMI, kg/m^2^, mean (SD)	Smoking, n (%)	Type of anesthesia	Procedure performed	Main findings
Kostroglou 2021^[21]^	Cohort study	Dexmedetomidine & Fentanyl	28	72 (11)	18 (64.28)	26 (5)	14 (50)		MT	Dexmedetomidine did not enhance the PaO₂/FiO₂ ratio during MT; however, it was linked to a lesser decline in FEV₁ postprocedure, decreased reliance on additional sedatives and analgesics, and expedited patient recovery.
		Midazolam/Fentanyl	27	67 (8)	17 (62.96)	28 (6)	14 (52)	Local anesthesia		
Lee 2016^[27]^	RCT	Dexmedetomidine	25	68.4 (6.4)	12 (48)	22.3 (2.7)	12 (48)	General anesthesia	VATS	For individuals with moderate COPD following lung cancer surgery, dexmedetomidine may improve lung function and oxygenation.
		Control	25	69.4 (8.7)	11 (44)	22.7 (2.1)	10 (40)			
Lee 2016^[22]^	RCT	Dexmedetomidine	25	62.0 (10.5)	26 (52)	23.6 (0.4)	15 (30)	General anesthesia	VATS	Intraoperative dexmedetomidine may improve postoperative outcomes and shorten hospital stay after VATS.
		Control	25	62.0 (11.5)	23 (46)	23.6 (0.4)	16 (32)			
Sirohiya 2022^[23]^	RCT	Dexmedetomidine	30	49.2 (17.8)	26 (86.67)	NA	NA	Local anesthesia	MT	Dexmedetomidine may provide superior sedation to midazolam during medical thoracoscopy.
		Midazolam	30	50.9 (16.1)	22 (73.33)	NA	NA			
Wang 2016^[24]^	RCT	Oxycodone	40	55.63 (11.20)	20 (50)	22.10 (2.13)	NA		VATS	Compared to oxycodone alone, oxycodone plus dexmedetomidine in PCA following VATS lobectomy may decrease opioid consumption, enhance pain management and patient satisfaction, and lessen the risk of nausea and vomiting.
		Dexmedetomidine & Oxycodone	40	54.25 (9.98)	20 (50)	21.93 (2.12)	NA	General anesthesia		
Wang 2020^[25]^	RCT	Dexmedetomidine	46	56.78 (12.81)	17 (36.95)	22.09 (3.22)	NA		VATS	Dexmedetomidine mitigated surgical stress and postoperative pain in moderate-intensity VATS lobectomy by attenuating noxious stimulation during general anesthesia.
		Normal saline	44	60.48 (12.58)	22 (50)	22.89 (2.85)	NA	General anesthesia		
Wu 2018^[26]^	RCT	Dexmedetomidine	30	59.0 (8.8)	16 (53.3)	NA	NA	General anesthesia	VATS	Dexmedetomidine mitigates inflammation and lung injury during one-lung ventilation by inhibiting the recruitment of alveolar neutrophils in thoracoscopic surgery.
			30	58.7 (10.1)	15 (50)	NA	NA			
		Normal saline								

COPD = chronic obstructive pulmonary disease, FiO_2_ = the fraction of inspired oxygen, FEV1 = forced expiratory volume in 1 s, NA = not applicable, MT = medical thoracoscopy, PCA = patient-controlled analgesia, PaO_2_ = partial pressure of oxygen in arterial blood, RCT = randomized clinical trial, VATS = video-assisted thoracoscopic surgery.

### 3.2. Outcomes

#### 3.2.1. Pain assessment

Five studies (n = 335) evaluated pain scores.^[21,23–25,27]^ Dexmedetomidine consistently demonstrated lower pain scores compared to control agents. In Sirohiya et al,^[23]^ patients receiving dexmedetomidine had significantly lower FACES pain scores (mean difference: 1.3), likely exceeding the minimum clinically important difference (MCID) of 1–1.5 points. Similarly, Kostroglou et al^[21]^ reported a 1.5-point reduction in NRS (numeric rating scale) scores when dexmedetomidine was added to midazolam/fentanyl, also surpassing the MCID threshold. In VATS studies, Lee et al (2016b)^[27]^ observed a 2-point difference in NRS scores, further supporting the clinical benefit of dexmedetomidine.

Wang et al (2016)^[24]^ found significantly higher patient satisfaction and lower visual analog scale scores at several postoperative timepoints with dexmedetomidine and oxycodone versus oxycodone alone, including a 1-point difference at 4 hours postop–meeting the lower bound of the MCID. In contrast, Wang et al (2020)^[25]^ showed a statistically significant difference in post-anesthesia care unit (PACU) NRS scores between dexmedetomidine and saline (0.58 points), but this did not meet the MCID threshold, suggesting limited clinical relevance. More details are shown in Table 2.

**Table 2:** Results of pain assessment.

Study ID	Intervention	Drugs used	Scale used	Time points of pain assessment	Results	*P*-value
Kostroglou 2021^[21]^	MT	DEX intravenous infusion supplemented by MZ/F vs MZ/F	NRS pain scores	Immediately postoperatively	NRS pain scores were lower in the DEX + MZ/F group (mean (95% CI): 1 (0.33–1.67)) compared to the MZ/F group (mean (95% CI): 2.54 (1.5–3.6).	.02
Lee 2016^[27]^	VATS	DEX	NRS pain scores	Multiple time points: • 10 min after arrival in the PACU • 30 min after arrival in the PACU	NRS pain scores were lower in the DEX group (median (IQR)): 3 (0–6) compared to the control group (median (IQR)): 5 (2–7).	.014
Sirohiya 2022^[23]^	MT	DEX vs MZ	Patient-rated FACES pain scale scores	Two hours postoperatively	Patient-rated face pain scale scores were lower in the DEX group (mean ± SD; 2.9 ± 1.8) compared to the MZ (mean ± SD; 4.2 ± 2.3).	.019
Wang 2016^[24]^	VATS	Oxycodone plus DEX vs oxycodone	VAS score	Multiple time points: • 4 h after surgery • 6 h after surgery • 24 h after surgery • 48 h after surgery	The level of patient satisfaction with pain management in the OD group was significantly higher than in the O group	<.001
Wang 2020^[25]^	VATS	DEX vs normal saline	NRS pain scores	Multiple time points: • At discharge from the PACU • 24 h after surgery • 48 h after surgery	The NRS pain scores in the normal saline group were higher than in the dexmedetomidine group at PACU discharge (3.25 ± 1.37 vs 2.67 ± 0.82, respectively).	.018

DEX = dexmedetomidine, F = fentanyl, MT = medical thoracoscopy, MZ = midazolam, NRS = the numeric rating scale, NA = not applicable, O group = oxycodone alone, OD group = oxycodone and dexmedetomidine, PACU = post-anesthesia care unit, SD = standard deviation, VAS = visual analogue scale, VATS = video-assisted thoracoscopic surgery.

#### 3.2.2. Lengths of hospital and care unit stay

Three studies reported the length of hospital stay.^[22,26,27]^ Only Lee et al (2016b)^[27]^ found a significant reduction in hospital stay with dexmedetomidine (median difference: 1.7 days, *P* = .045). Other studies, including Lee et al (2016a)^[22]^ and Wu et al (2018),^[26]^ reported no significant differences.

Regarding care unit stay, 2 studies evaluated the duration of PACU or intensive care unit (ICU). Wang et al (2016)^[24]^ reported no difference in PACU time. Wu et al (2018)^[26]^ observed shorter ICU stay in the dexmedetomidine group (0.2 vs 0.8 days), though the difference was not statistically significant (*P* = .11). More details are shown in Table 3.

**Table 3:** Results of lengths of care units and hospital stay.

Study ID	Intervention	Drugs used	Care unit	Results	*P*-value
*Length of care unit stay (d*)					
Wang 2016^[24]^	VATS	Oxycodone plus DEX vs oxycodone	PACU	The length of PACU stay did not differ significantly between the OD group, mean (SD) 21.98 (4.08), and the O group, mean (SD) 21.63 (3.71).	.567
Wu 2018^[26]^	VATS	DEX vs saline	ICU	Shorter length of ICU stay was reported in the Dex group compared to the saline group; mean ± SD (0.2 ± 0.6 vs 0.8 ± 1.7 d).	.1152
*Length of hospital stay (d*)					
Lee 2016^[22]^	VATS	DEX	Hospital	The duration of hospital stay did not differ between groups. DEX group: median (IQR) 6.5 (5–10) compared with the Control group: median (IQR) 7.1 (6–11).	.102
Lee 2016^[27]^	VATS	DEX	Hospital	The length of hospital stay was significantly shorter in the DEX group; median (IQR) 6.7 (3–9) compared with the Control group; median (IQR) 8.4 (4–9).	.045
Wu 2018^[26]^	VATS	DEX vs saline	Hospital	Length of hospital stays showed an insignificant difference between both groups; DEX group (mean ± SD; 5.6 ± 2.5 dcompared to saline (mean ± SD; 5.9 ± 3.1 d).	.6491

DEX = dexmedetomidine, ICU = intensive care unit, IQR1 = interquartile range, NA = not applicable, O group = oxycodone alone, OD group = oxycodone and dexmedetomidine, PACU = post-anesthesia care unit, SD = standard deviation, VATS = video-assisted thoracoscopic surgery.

#### 3.2.3. Complications

Medical complications were generally infrequent. One MT study (Sirohiya et al)^[23]^ reported hypotension in one patient with dexmedetomidine and 2 with midazolam (*P* = .05). Three VATS studies reported hypotension and bradycardia combined; differences were not statistically significant. Kostroglou et al^[21]^ noted no such events. Pulmonary complications, such as pneumonia, were rare and comparable between groups, with one or 2 events reported per study arm.^[26,27]^

Surgical complications were also uncommon. Wu et al (2018)^[26]^ reported slightly higher frequencies of air leaks and subcutaneous emphysema in the dexmedetomidine group, but none reached statistical significance. Chylothorax was reported in 2 studies, with no cases in the dexmedetomidine groups and one case each in the control arms. Complete complication data are available in Tables 4 and 5.

**Table 4:** Results of medical complications.

Study ID	Intervention	Drugs used	Results	*P*-value
*Hypotension*				
Kostroglou 2021^[21]^	MT	DEX intravenous infusion supplemented by MZ/F vs MZ/F	No incidence of hypotension was reported in the 2 groups.	NA
Lee 2016^[27]^	VATS	DEX	Hypotension was observed in 2 patients in the DEX group (50) and zero patients in the control group (50).	.495
Sirohiya 2022^[23]^	MT	DEX vs MZ	Hypotension was observed in 1 patient in the DEX group (30) and 2 patients in the MZ group (30).	.500
Wang 2016^[24]^	VATS	Oxycodone plus DEX vs oxycodone	No hypotension was observed in either group.	NA
*Bradycardia*				
Kostroglou 2021^[21]^	MT	DEX intravenous infusion supplemented by MZ/F vs MZ/F	No cases of bradycardia were reported with either group.	NA
Lee 2016^[27]^	VATS	DEX	Bradycardia was observed in 2 patients in the dexmedetomidine group (50) and zero patients in the control group (50).	.495
Wang 2016^[24]^	VATS	Oxycodone plus DEX vs oxycodone	No patient developed bradycardia in both groups. However, the HR in the OD group tended to be lower than in the O group. So, dexmedetomidine is contraindicated for patients with bradycardia or a heart blockage.	NA
*Pneumonia*				
Lee 2016^[27]^	VATS	DEX	Pneumonia was observed in 1 patient in the dexmedetomidine group (50) and 2 patients in the control group (50).	> .99
Wu 2018^[26]^	VATS	DEX vs saline	Pneumonia occurred in zero patients in the dexmedetomidine group (30) and 1 patient in the saline group (30).	.3198

DEX = dexmedetomidine, F = fentanyl, MT = medical thoracoscopy, MZ = midazolam, NA = not applicable, O group = oxycodone alone, OD group = oxycodone and dexmedetomidine, VATS = video-assisted thoracoscopic surgery.

**Table 5:** Results of surgical complications.

Chylothorax				
Study ID	Intervention	Drug used	Results	*P*-value
*Chylothorax*				
Lee 2016^[27]^	VATS	DEX	Chylothorax was observed in zero patients in the DEX group (50) and 1 patient in the control group (50).	>.99
Wu 2018^[26]^	VATS	DEX vs Saline	Chylothorax occurred in 1 patient in the DEX group (30) and 2 patients in the saline group (30).	.5491
*Subcutaneous* emphysema				
Wu 2018^[26]^	VATS	DEX vs Saline	Subcutaneous emphysema occurred in 8 patients in the DEX group (30) and 2 patients in the saline group (30).	.0395
*Air* leaks need pleurodesis				
Wu 2018^[26]^	VATS	DEX vs Saline	Air leaks need pleurodesis occurred in 3 patients in the DEX group (30) and 1 patient in the saline group (30).	.3017

DEX = dexmedetomidine, VATS = video-assisted thoracoscopic surgery.

### 3.3. Narrative synthesis

Due to substantial clinical and methodological heterogeneity, including variation in sedation protocols, dosing regimens, outcome definitions, and comparator agents, a meta-analysis was not feasible. This review instead presents a systematic review without meta-analysis, with descriptive synthesis for each outcome. These findings underscore the need for additional standardized, high-quality trials to enable robust comparisons of sedation strategies in interventional chest procedures.^[19,20]^

## 4. Discussion

This systematic review evaluated the safety and efficacy of dexmedetomidine as a sedative agent during interventional chest procedures, specifically MT and VATS. Across the included studies, dexmedetomidine was associated with improved analgesic outcomes compared to midazolam or other control agents, including saline, midazolam with or without fentanyl, and oxycodone.

In procedures typically performed under procedural sedation, such as MT, dexmedetomidine consistently demonstrated clinically meaningful reductions in pain scores. In Sirohiya et al,^[23]^ the reduction in patient-reported pain exceeded the MCID, supporting its analgesic superiority over midazolam. Similarly, Kostroglou et al^[21]^ found that dexmedetomidine added to midazolam/fentanyl significantly lowered pain scores, also exceeding the MCID threshold. These findings reinforce dexmedetomidine’s role in procedures performed under conscious sedation, particularly in spontaneously breathing patients.

In VATS, which is conducted under general anesthesia, 3 studies showed significantly lower postoperative pain scores in patients receiving intraoperative dexmedetomidine compared to control groups. In Lee et al (2016b)^[27]^ and Wang et al (2016),^[24]^ these reductions also met or exceeded MCID criteria, suggesting both statistical and clinical significance.

Findings regarding lengths of stay were mixed. While Lee et al (2016b)^[27]^ reported a significantly shorter hospital stay with dexmedetomidine, other studies did not observe meaningful differences in either hospital or ICU lengths of stay. Only one study showed a reduced ICU stay, while PACU durations were largely comparable.

Medical and surgical complications were generally uncommon across the included studies. Hypotension and bradycardia were reported in a few VATS studies, but differences between dexmedetomidine and control groups were not statistically significant. One MT study noted isolated cases of hypotension with both dexmedetomidine and midazolam. Pulmonary complications, such as pneumonia, were rare and occurred at similar rates between groups. Surgical complications, including air leaks, subcutaneous emphysema, and chylothorax, were infrequent and did not differ significantly across treatment arms.

A prior systematic review by Feray et al (2022)^[28]^ recommended the intraoperative use of intravenous dexmedetomidine, citing its benefits in pain control, sedation quality, and postoperative outcomes. Our findings are consistent with this evidence, as the included studies demonstrated that dexmedetomidine significantly reduced pain scores compared to midazolam, particularly in procedures such as VATS and MT. Feray et al^[28]^ also reported that dexmedetomidine reduced postoperative restlessness, cognitive impairment, nausea, and vomiting, while enhancing pulmonary function. However, they noted an increased incidence of bradycardia and hypotension, effects that were not clinically significant in their analysis but warrant caution in patients with preexisting cardiac conditions. They also advised against routine postoperative use due to inconsistent supporting evidence.

These findings underscore the need for tailored sedation strategies in interventional chest procedures, taking into account both the procedural setting and patient risk factors. MT is typically performed under procedural sedation in spontaneously breathing patients, while VATS is conducted under general anesthesia.^[29–31]^ These differences impact not only the pharmacologic demands of sedation but also the interpretation of outcomes such as respiratory depression and hemodynamic stability.

Dexmedetomidine, with its minimal respiratory suppression and intrinsic analgesic effects, appears especially suitable for non-intubated procedures like MT. However, its known cardiovascular effects, particularly bradycardia and hypotension, require careful monitoring, especially in settings where continuous hemodynamic support is less readily available. In contrast, midazolam, while well-tolerated, may offer less optimal analgesia and a slower recovery profile, which may limit its use in certain procedural contexts.^[29–31]^

These results support the importance of individualized sedation planning, emphasizing the need for thorough preprocedural assessments, appropriate monitoring, and dose titration strategies. Future research should aim to refine patient selection, standardize sedation protocols, and better define the balance between efficacy and safety, particularly in high-risk populations undergoing MT or VATS.

### 4.1. Strengths and limitations of the study

This systematic review provides a comprehensive synthesis of the current evidence on the safety and efficacy of dexmedetomidine in interventional chest procedures, offering valuable insight into sedation practices during VATS and MT. The findings particularly emphasize the analgesic advantages of dexmedetomidine compared to midazolam and other control agents, with several studies reporting clinically meaningful reductions in pain scores.

However, the study is not without limitations. A major constraint was the heterogeneity across the included studies, which varied in terms of dosing protocols, outcome definitions, and patient characteristics. This variability precluded the possibility of conducting a meta-analysis and limited the comparability of results. Furthermore, some conclusions, such as those related to ICU stay duration or specific complications, were based on isolated findings from single studies, highlighting the need for further validation.

An additional limitation lies in the narrow scope of outcome reporting within the available literature. Key clinical indicators such as respiratory depression, hemodynamic interventions, sedation depth, and satisfaction from either patients or clinicians were either absent or inconsistently documented, restricting our ability to evaluate these dimensions. This gap underscores the pressing need for more standardized reporting and comprehensive outcome measures in future primary studies to enable more robust and clinically applicable conclusions.

### 4.2. Future perspectives

Our systematic review underscores the promise of dexmedetomidine as an effective sedative agent for interventional chest procedures, particularly in managing procedural pain. However, further research is needed to better define its clinical role and guide optimal use. Future studies should involve larger, multicenter RCTs that utilize standardized sedation protocols and clearly defined outcome measures across diverse patient populations. It is also important to examine dexmedetomidine’s broader clinical impact, including its effects on patient comfort, cough suppression, and overall procedural success, not just pain reduction.

Additionally, more work is required to assess dexmedetomidine’s long-term safety and potential benefits, especially in terms of cognitive outcomes and pulmonary function. Economic evaluations comparing dexmedetomidine to other sedative options are also warranted, taking into account medication costs, monitoring requirements, and potential reductions in hospital length of stay. By addressing these knowledge gaps, future research will contribute to a more comprehensive understanding of dexmedetomidine’s role in thoracic procedures and support more informed, evidence-based decisions that enhance patient outcomes and procedural efficiency.

## 5. Conclusion

In conclusion, this systematic review highlights dexmedetomidine as a promising sedative agent for MT and VATS. It offers effective pain control, may reduce ICU and hospital stay in some settings, and maintains respiratory stability better than traditional agents. Nonetheless, its use must be individualized, especially in patients with cardiovascular comorbidities. Further high-quality studies are warranted to better define its optimal dosing, safety profile, and role in diverse patient populations.

## Author contributions

**Conceptualization:** Nada M. Al-Dardery.

**Investigation:** Moaz M. El-Sayed.

**Methodology:** Nada M. Al-Dardery, Mohamed El-Samahy.

**Project administration:** Kalpana Singh.

**Software:** Mohamed El-Samahy.

**Supervision:** Abdulqadir J. Nashwan.

**Visualization:** Moaz M. El-Sayed, Kalpana Singh.

**Writing – original draft:** Abdulrhman M. Khaity, Nada M. Al-Dardery, Mohamed El-Samahy, Moaz M. El-Sayed.

**Writing – review & editing:** Abdulrhman M. Khaity, Sheikh M. Jamal, Abdulqadir J. Nashwan.

## Supplementary Material


