# Supplementary material for: Safety and efficacy of dexmedetomidine in interventional chest procedures: A systematic review
Source: Medicine (Baltimore). 2025 Aug 15;104(33):e43911. doi: 10.1097/MD.0000000000043911 (PMC12367014; doi:10.1097/MD.0000000000043911)
Supplement: Supplementary file 1 [file medi-104-e43911-s001.docx]

| ***Study ID*** | ***Cohort studies*** | | | | | | | | |
| --- | --- | --- | --- | --- | --- | --- | --- | --- | --- |
|  | ***Selection*** | | | | ***Comparability*** | ***Outcome*** | | | ***Quality Score*** |
|  | ***Representativeness of the exposed cohort*** | ***Selection of the non-exposed cohort*** | ***Ascertainment of exposure*** | ***Demonstration that outcome of interest was not present at start of study*** | ***Comparability of cohorts on the basis of the design or analysis*** | ***Assessment of outcome*** | ***Was follow-up long enough for outcomes to occur*** | ***Adequacy of follow up of cohorts*** |  |
| *Kostroglou 2021*[^1^](https://sciwheel.com/work/citation?ids=17242330&pre=&suf=&sa=0&dbf=0) |  | *** | *** | *** | **** | *** | *** | *** | *Good* |

***Supplement Table 1.*** Assessment risk of bias of observational study using NOS

**References**

[1.    Kostroglou A, Kapetanakis EI, Matsota P, et al. Monitored Anesthesia Care with Dexmedetomidine Supplemented by Midazolam/Fentanyl versus Midazolam/Fentanyl Alone in Patients Undergoing Pleuroscopy: Effect on Oxygenation and Respiratory Function. *J Clin Med*. 2021;10(16). doi:10.3390/jcm10163510](https://sciwheel.com/work/bibliography/17242330)
